# Harnessing extracellular vesicles using liquid biopsy for cancer diagnosis and monitoring: highlights from AACR Annual Meeting 2024

**DOI:** 10.1186/s13045-024-01577-y

**Published:** 2024-07-29

**Authors:** Xinming Su, Zeping Shan, Shiwei Duan

**Affiliations:** 1 Key Laboratory of Novel Targets and Drug Study for Neural Repair of Zhejiang Province, School of Medicine, Hangzhou City University, Hangzhou, Zhejiang China; 2 Department of Clinical Medicine, Hangzhou City University, Hangzhou, Zhejiang China

**Keywords:** Liquid biopsy, Extracellular vesicles, Cancer diagnosis, Biomarkers, Precision medicine

## Abstract

Liquid biopsy, an advanced technology for analyzing body fluid samples, is gaining traction in cancer diagnostics and monitoring. Blood-based liquid biopsy, particularly focusing on cell-free DNAs (cf-DNAs), circulating tumor cells (CTCs), and extracellular vesicles (EVs), has garnered significant attention. EVs stand out for their potential in tumor diagnosis, prognosis prediction, and treatment response assessment, owing to their stable molecular cargo and clear extraction process. At the recent American Association for Cancer Research (AACR) Annual Meeting 2024, groundbreaking EVs-based liquid biopsy studies showcased promising strides in early detection and diagnosis of various cancers, including breast cancer (BC), high-grade serous ovarian cancer (HGSOC), pancreatic ductal adenocarcinoma (PDAC), colorectal cancer (CRC), colon adenocarcinoma (COAD), head and neck cancer (HNC), neuroblastoma, and retinoblastoma (RB). Despite these advancements, challenges persist in translating EVs biomarkers into clinical practice. Overcoming these challenges promises to propel EVs-based liquid biopsy into a new era of personalized precision medicine, revolutionizing cancer detection, monitoring, and treatment.

**Supplementary Information:**

The online version contains supplementary material available at 10.1186/s13045-024-01577-y.

To the Editor,

Currently, blood-based liquid biopsy dominates research efforts, primarily detecting free blood components, including cell-free DNAs (cf-DNAs), circulating tumor cells (CTCs), and extracellular vesicles (EVs). EVs are vesicles released by cells into the extracellular environment. They have shown high accuracy and sensitivity in early cancer detection, classification, and treatment evaluation, making them valuable as a source of biomarkers. They carry stable and representative molecular components, including proteins, nucleic acids, and lipids, enhancing their clinical utility. Excitingly, The American Association for Cancer Research (AACR) Annual Meeting 2024 highlighted several EVs-based liquid biopsies, heralding significant progress in early detection and diagnosis of common malignancies like breast cancer (BC), high-grade serous ovarian cancer (HGSOC), pancreatic ductal adenocarcinoma (PDAC), colorectal cancer (CRC), colon adenocarcinoma (COAD), head and neck cancer (HNC), neuroblastoma, and retinoblastoma (RB) (Tables 1 and 2).

**Table 1:** Sample types and EV biomarker extraction methods used for liquid biopsy

Indication	Biomarker type	Sample	Detection technology	Application	Reference
BC	miRNA	plasma	IAC	diagnosis	[1]
BC	miRNA	cyst fluid	membrane affinity binding technique, EP, microfluidics, and RT-qPCR	monitor	[2]
TNBC	protein	plasma	microfluidic technologies	diagnosis	[3]
HGSOC	protein	serum	LC-MS/MS, PEA, and ELISA	diagnosis	[4]
HGSOC	DNA	ascite	WGS, SNP array, and ddPCR	diagnosis	[5]
HGSOC	protein	plasma	RNA sequencing	diagnosis	[6]
CRC	protein	plasma	qPCR	diagnosis	[7]
CRC	protein	plasma	ECL	monitor	[8]
COAD	protein	plasma	LC-MS/MS and ELISA	diagnosis	[9]
PDAC	miRNA and glycoprotein	plasma	RT-qPCR	diagnosis	[10]
PDAC	lipid, metabolite and protein	plasma	MS	diagnosis	[11]
PDAC	protein	serum	ExoView™ platform	monitor	[12]

BC, Breast cancer; COAD, Colon adenocarcinoma; CRC, Colorectal cancer; ddPCR, Droplet digital polymerase chain reaction; ECL, Electrochemiluminescence; EP, Electrophoresis; ELISA, Enzyme linked immunosorbent assay; HGSOC, High-grade serous ovarian cancer; IAC, Immune affinity capture; LC-MS/MS, Liquid chromatograph mass spectrometer/Mass spectrometer; PDAC, Pancreatic ductal adenocarcinoma; PEA, Proximity extension assay; qPCR, Quantitative polymerase chain reaction; RT-qPCR, Reverse transcription-quantitative polymerase chain reaction; SNP, Single nucleotide polymorphism; TNBC, Triple-negative breast cancer; WGS, Whole genome sequencing

**Table 2:** Specific targets, assay cohort, and assay performance of liquid biopsy

Indication	Target	Patient type and number	AUC	Sensitivity	Specificity	Reference
BC	EV-miRNAs (miR-21, miR-106b, miR-181a, miR-484, and miR-1260b)	BC patients (*N* = 120), individuals with benign tumors (*N* = 46), and healthy controls (*N* = 45)	> 0.8	0.5714	0.95	[1]
BC	EV-miRNAs (miR-607, miR-202-3p, miR-6872-3p, miR-769-3p, miR-5195-3p, miR-4443, miR-4713-3p, miR-6076, and miR-4515) and clinical variables (menopausal status, familiarity, and type of cyst yielded)	GCDB patients (*N* = 58) and healthy controls (*N* = 59)	0.73 (6 EV-miRNAs), 0.8 (3 EV-miRNAs and 3 clinical variables), and 0.8 (2 EV-miRNAs and 2 clinical variables)	/	/	[2]
TNBC	ECM1, MBL2, and BTD	BC patients and healthy controls (SUM = 30)	/	0.933	0.93	[3]
HGSOC	EV protein (CFH, PCP, CCNE1, and CA-125)	HGSOC patients and healthy controls (SUM = 250)	0.94 (CFH), 0.91 (PCP), 0.83 (CCNE1), and 0.42 (MUC16 (CA-125))	/	/	[4]
HGSOC	BST2, MUC1, and sTn	HGSOC patients (*N* = 58, 17 Stage I, 30 Stage II, and 10 Stage III) and benign ovarian tumor (*N* = 17)	/	/	/	[5]
HGSOC	Copy numbers of RAD51, BRCA1, AKT2, CCNE1, and MSH6	HGSOC patients and cell lines (SUM = 124)	/	/	/	[6]
CRC	RNF208	CRC patients (*N* = 39) and healthy controls (*N* = 16)	/	/	/	[7]
CRC	CEA and CD73	/	0.975	/	/	[8]
COAD	7 EV proteins	COAD patients (*N* = 84) and healthy controls (*N* = 20)	0.913, 1.000, 0.985, and 0.984 (for Stages I through IV)	/	/	[9]
PDAC	cf-miRNAs, ex-miRNAs, and CA19-9	Japan (PAAD patients (*N* = 150) and healthy controls (*N* = 102)); the United States (PAAD patients (*N* = 139) and healthy controls (*N* = 193)); South Korea (PAAD patients (*N* = 184) and healthy controls (*N* = 86)); China (PAAD patients (*N* = 50) and healthy controls (*N* = 80))	0.97	0.95	0.96	[10]
PDAC	5-lipids, 2-metabolites, and 5-proteins	early-stage PC patients (*N* = 60), pancreatitis (*N* = 39), precursor lesions of pancreas (*N* = 45), and healthy controls (*N* = 50)	> 0.95	> 0.9	> 0.9	[11]
PDAC	ALPPL2 and THBS2	PDAC patients (*N* = 26)	/	/	/	[12]

BC, Breast cancer; CEA, Carcinoembryonic antigen; cf-miRNAs, Cell-free microRNAs; COAD, Colon adenocarcinoma; CRC, Colorectal cancer; ex-miRNAs, Extracellular microRNAs; EV, Extracellular vesicle; GCDB, Gross cyst disease of the breast; HGSOC, High-grade serous ovarian Cancer; PAAD/PC, Pancreatic cancer; PDAC, Pancreatic ductal adenocarcinoma; TNBC, Triple-negative breast cancer

BC is one of the most prevalent and lethal cancers affecting women worldwide. Jee Ye Kim et al. used BC-derived EVs from blood samples, identifying 5 EV-miRNAs as potential biomarkers, with AUC values exceeding 0.8 [1]. In another case-control study, Barbara Cardinali et al. used cyst fluid from patients and extracted 7 EV-miRNAs with clinical variables into a risk model, yielded an AUC of 0.8 [2]. Additionally, they integrated machine learning to analyze the EV proteomic data, discovered that 3 proteins can be combined to effectively distinguish TNBC patients from healthy individuals, achieving 93.3% sensitivity and 93% specificity [3].

Ovarian cancer (OC) is a prevalent gynecological malignancy, with HGSOC being its most aggressive subtype. Michelle Lightfoot et al. verified 4 EV-contained proteins serving as potent biomarkers for late-stage HGSOC detection, with AUC values of 0.94 (CFH), 0.83 (CCNE1), 0.42 (MUC16), and 0.91 (PCP) [4]. Multi-target biomarkers showed superior predictive efficacy, achieving a true positive rate (TPR) of 0.943 and a false positive rate (FPR) of 0.000 in 70 patients [4]. It is worth noting that another team determined the co-localization of tumor markers BST2, MUC1, and sTn on EVs, demonstrating their effectiveness in the early detection of HGSOC [5]. Additionally, Ryosuke Uekusa et al. innovatively revealed that copy number variation (CNV) status of RAD51, BRCA1, AKT2, CCNE1, and MSH6 in malignant tumor tissues were significantly higher than those in benign tissues [6].

CRC is a leading malignant tumor of the digestive system globally. Guo et al. conducted bioinformatics analyses using public databases along with qPCR results from clinical samples, founded that the EV protein RNF208 is a novel diagnostic tool for CRC [7]. Another team enhanced the accuracy of the standard soluble carcinoembryonic antigen (CEA) by incorporating specific EVs, achieving an AUC of 0.975 [8]. Colorectal adenocarcinoma (COAD) is a significant component of the global cancer disease spectrum. Yura Seo et al. identified 7 EV proteins as combined biomarkers. The AUC values for COAD stages I to IV were 0.913, 1.000, 0.985, and 0.984, respectively [9].

Pancreatic cancer (PC) is a highly malignant tumor of the digestive tract, with PDAC comprises about 90% of all PCs. Xu et al. developed a diagnostic model using a combination of biomarkers (including 3 types of miRNAs and CA19-9), with an AUC of 0.97, sensitivity of 0.95, and specificity of 0.96 [10]. Meanwhile, Shivani Bansal et al. introduced a classification algorithm based on 12 multi-omics analytes within EVs, demonstrated high accuracy, sensitivity and specificity in PC identification and stratification [11]. In addition, related study found that the concentrations of ALPPL2^+^ and THBS2^+^ EVs in PDAC patients were significantly increased and closely correlated with changes in tumor size [12].

Furthermore, in certain rare cancers, including HNC, neuroblastoma, and RB, EV-based liquid biopsy demonstrates significant potential. This approach enhances diagnostic and monitoring accuracy and offers innovative sample types and methods (see Supplementary Material).

In summary, EV-based liquid biopsy methods show great potential for the early diagnosis of cancer, allowing for the differentiation between benign and malignant tumors and between tumor grades. Additionally, these methods are effective in monitoring cancer progression and evaluating patients’ responses to treatment. It is imperative to continuously promote relevant basic and clinical research on liquid biopsy based on EV biomarkers while concurrently establishing industrialization and commercialization systems along with corresponding policies.

### Electronic supplementary material

Below is the link to the electronic supplementary material.

Supplementary Material 1

## Data Availability

No datasets were generated or analysed during the current study.

## Abbreviations

AACR: The American Association for Cancer Research
AUC: Area under curve
BC: Breast cancer
CEA: Carcinoembryonic antigen
cf-DNAs: Cell-free DNAs
cf-miRNAs: Cell-free microRNAs
CNV: Copy number variation
COAD: Colon adenocarcinoma
CRC: Colorectal cancer
CTCs: Circulating tumor cells
EVs: Extracellular vesicles
ex-miRNAs: Extracellular microRNAs
FPR: False positive rate
HGSOC: High-grade serous ovarian cancer
HNC: Head and neck cancer
OC: Ovarian cancer
PC: Pancreatic cancer
PDAC: Pancreatic ductal adenocarcinoma
RB: Retinoblastoma
TNBC: Triple-negative breast cancer
TPR: True positive rate
