# Supplementary material for: Harnessing extracellular vesicles using liquid biopsy for cancer diagnosis and monitoring: highlights from AACR Annual Meeting 2024
Source: J Hematol Oncol. 2024 Jul 29;17:55. doi: 10.1186/s13045-024-01577-y (PMC11287960; doi:10.1186/s13045-024-01577-y)
Supplement: Supplementary file 1 — Supplementary Material 1 [file 13045_2024_1577_MOESM1_ESM.docx]

**Harnessing Extracellular Vesicles using Liquid Biopsy for Cancer Diagnosis and Monitoring: Highlights from AACR Annual Meeting 2024**

**Authors:** Xinming Su^1,2^, Zeping Shan^1,2^, Shiwei Duan^1,2,*^

1. Key Laboratory of Novel Targets and Drug Study for Neural Repair of Zhejiang Province, School of Medicine, Hangzhou City University, Hangzhou, Zhejiang, China.

2. Department of Clinical Medicine, Hangzhou City University, Hangzhou, Zhejiang, China.

*: Correspondence should be addressed to Dr. Shiwei Duan (duansw@hzcu.edu.cn).

**Supplementary Materials**

EV’s excellent applicability for diagnosis and monitoring in other rare cancers

(Page 2)

**Table S1**. Sample types and EV biomarker extraction methods used by cancer liquid biopsy in other rare cancers

(Page 3)

**Table S2**. Cancer liquid biopsy's specific targets, assay cohort, and assay performance in other rare cancers

(Page 4)

**References**

(Page 5)

**EV’s excellent applicability for diagnosis and monitoring in other rare cancers**

EVs have also shown excellent applicability in other rare cancers. Randy P. Carney et al. analyzed EVs from biological fluids such as saliva and plasma using Raman spectroscopy (RS), then integrated machine learning modules to improve the detection accuracy of HNC (1). Neuroblastoma, one of the most common childhood cancers (2), was further investigated by Gianluca Sala et al., who demonstrated that EV-related LGALS3BP can serve as a key biomarker in neuroblastoma liquid biopsy (3). Retinoblastoma (RB), another common ocular malignancy in children that cannot be biopsied (4), was studied by Anne Amacker et al. They innovatively used aqueous humor (AH) as a sample source of EVs in liquid biopsy and verified the significant effectiveness of CD63, CD81, and CD133 in RB typing through Macsplex, a multiplex bead-based flow cytometry assay (5).

**Table S1. Sample types and EV biomarker extraction methods used by cancer liquid biopsy in other rare cancers**

| **Indication** | **Biomarker type** | **Sample** | **Detection technology** | **Application** | **Reference** |
| --- | --- | --- | --- | --- | --- |
| HNC | / | saliva and plasma | RS and SERS | diagnosis | (1) |
| Neuroblastoma | protein | / | ELISA and confocal microscopy | diagnosis | (3) |
| RB | protein | AH | Macsplex | diagnosis | (5) |

AH, Aqueous humor; ELISA, Enzyme linked immunosorbent assay; GLC, congenital glaucoma; HNC, Head and neck cancer; RB, Retinoblastoma; RS, Raman spectroscopy; SERS, Surface enhanced Raman spectroscopy.

**Table S2. Cancer liquid biopsy's specific targets, assay cohort, and assay performance in other rare cancers**

| **Indication** | **Target** | **Patient type and number** | **AUC** | **Sensitivity** | **Specificity** | **Reference** |
| --- | --- | --- | --- | --- | --- | --- |
| HNC | / | HNC patients and healthy controls (SUM>100) | / | / | / | (1) |
| Neuroblastoma | LGALS3BP | / | / | / | / | (3) |
| RB | CD9, CD63 and CD81 | GLC patients (N=2) and RB patients (N=5) | / | / | / | (5) |

GLC, congenital glaucoma; HNC, Head and neck cancer; RB, Retinoblastoma.

**References**

1. Carney RP, Navas-Moreno M, Birkeland A. Abstract 1067: Label free Raman spectroscopy of extracellular vesicles for early stage liquid biopsy cancer detection. Cancer Research. 2024;84(6_Supplement):1067-.

2. Matthay KK, Maris JM, Schleiermacher G, Nakagawara A, Mackall CL, Diller L, Weiss WA. Neuroblastoma. Nat Rev Dis Primers. 2016;2:16078.

3. Sala G, Capone E, Cela I, Lovato G, Lamolinara A, Iezzi M, et al. Abstract 2609: Vesicular LGALS3BP is a neuroblastoma biomarker and a therapeutic target for combination therapy with antibody-drug conjugate and checkpoint inhibitors. Cancer Research. 2024;84(6_Supplement):2609-.

4. Dimaras H, Kimani K, Dimba EA, Gronsdahl P, White A, Chan HS, Gallie BL. Retinoblastoma. Lancet. 2012;379(9824):1436-46.

5. Amacker A, Peng C-C, Reiser BJ, Berry JL, Xu L. Abstract 3642: Phenotypic biomarkers of aqueous humor extracellular vesicles from retinoblastoma eyes. Cancer Research. 2024;84(6_Supplement):3642-.
